# Surgical Complications Associated With Fasciectomy for Dupuytren's Disease: A 20-Year Review of the English Literature

**Published:** 2010-01-27

**Authors:** Keith Denkler

**Affiliations:** UCSF Divison of Plastic Surgery, 275 Magnolia Ave, Larkspur, CA 94939. Dr Denkler is in private practice also.

## Abstract

**Objective:** Excisional surgery is the mainstay of treatment of Dupuytren's disease. Although outcomes are generally good, complications are common. The objective of this study was to evaluate intraoperative and postoperative complications associated with fasciectomy for Dupuytren's disease. **Methods:** A literature search was conducted to identify published, original research that reported surgical complications associated with fasciectomy from 1988 to 2008. Search results were manually evaluated for relevance. Complication rates according to types of disease (primary or recurrent disease) and according to time (intraoperative vs postoperative) and type were collated. **Results:** A total of 143 articles were identified; 41 met inclusion criteria, and of these, 28 reported overall surgical complication rates ranging from 3.6% to 39.1%. Major complications occurred in 15.7%, including digital nerve injury 3.4%, digital artery injury 2%, infection 2.4%, hematoma 2.1%, and complex regional pain syndrome 5.5%. Other common, more minor injuries included flare reaction in 9.9%, wound healing complications in 22.9%, and a range of other complications. In the few (*n* = 3) studies in which primary and recurrent diseases were directly compared, digital nerve injuries and digital artery injuries were approximately 10 times more common in patients with recurrent disease (˜20%) than those with primary disease (˜2%), though the numbers are too small for statistical significance. **Conclusions:** A review of published reports by surgeons shows that surgical fasciectomy for Dupuytren's disease has a high number of complications. Surgeons should be mindful of the potential for intraoperative and postoperative complications and counsel their patients accordingly.

Dupuytren's disease was originally noted by Plater in 1614^1^ and carries the eponym of Baron Guillaume Dupuytren, who first lectured on the disease in 1831.^2^ Although Cline in 1777 and Cooper in 1822 had described the fascial contracture and its treatment by fasciotomy, they were not mentioned in Dupuytren's discussions.^1^ Dupuytren's disease is a genetic disorder of abnormal collagen production and deposition in the hand that is commonly characterized by metacarpophalangeal (MP) and proximal interphalangeal (PIP) joint contractures in the ring and little fingers. Dupuytren's disease can affect all races, but people of northern European descent are most commonly affected,^3^^-^5 with 3% to 6% of white adults acquiring the condition during their lifetime.^3^,^6^ Dupuytren's diathesis, which manifests as a more aggressive form of the disease, comprises a positive family history with 1 or more affected siblings or parents, male gender, age less than 50 years at onset, bilateral involvement, ectopic manifestations (particularly Garrod's pads), and Caucasian ethnicity.^7^ Furthermore, evidence indicates that Dupuytren's disease is more likely to occur in those with certain underlying conditions such as diabetes,^8^ thyroid disorders,^9^ alcoholism,^10^ and epilepsy.^3^ Lower incidences of Dupuytren's occur in those afflicted by rheumatoid arthritis.^11^

Genetic analyses show that Dupuytren's disease is an autosomal dominant disorder with variable penetrance and gene expression.^12^ Genetic predisposition, combined with diatheses, lifestyle choices, (eg, alcohol consumption), or trauma,^13^,^14^ can trigger micro ruptures of the collagen fibers of the palmar fascia, fibroblast proliferation, and differentiation of fibroblasts into myofibroblasts.^15^,^16^ The expanding fibroblast pool and excess collagen deposition cause nodule and cord formation in the palm or digits.

Dupuytren's disease is progressive, with onset typically occurring later in life and worsening over the course of several months to several years.^17^ In early stages, skin pitting and dimpling are commonly observed as pretendinous bands connected to the dermis begin to contract.^18^ Initially, nodules are painless and hand function is generally retained. However, as the disease progresses, cords begin to contract, causing finger flexion deformities and diminished hand function.^18^ The contractile properties of myofibroblasts are thought to cause the cords to shorten,^15^ resulting in the hallmark contractures that characterize Dupuytren's disease.

Few treatment options exist for those with Dupuytren's contracture. Surgery is currently the mainstay of treatment and is recommended for functionally impaired patients with MP joint contractures of more than 30°.^18-223^ Indications for the treatment of PIP joint contracture varies. Some authors recommend surgery for any degree of PIP contracture.^20^,^22^ Others feel that there should be approximately 15° (references 18, 24) or 30° (reference 25) of PIP contracture to warrant surgery. In contrast to these established guidelines, McGrouther asserts that it is better to “rely on functional difficulty and the rate of progression when deciding on surgery, rather than choosing a set amount of joint contracture.”^26(p167)^

Open, limited (subtotal) fasciectomy is the most commonly used surgical procedure,^10^,^27^^-^30 but open or closed fasciotomies, including percutaneous needle fasciotomy (ie, needle aponeurotomy), are also performed.^31^^-^35 Although surgery provides positive outcomes for most patients, extensive hand therapy is typically required after surgery. Not all patients with Dupuytren's contracture are candidates for surgery; advanced age, comorbidities, or both, often exclude patients from undergoing fasciectomy. In this circumstance, closed fasciotomy^26^,^36^ or needle aponeurtomy^35^ is often recommended. To date, no effective pharmacotherapy has been approved for the treatment of Dupuytren's disease,^37^ though an investigational procedure with *Clostridium histolyticum* collagenase (enzymatic fasciotomy) shows promise.^38^

Dupuytren's disease is not curable because it is a genetic disease and has a cellular basis. Surgeons can help improve hand impairment due to Dupuytren's disease by surgical techniques. These corrective surgical procedures improve hand function for most patients; however, intraoperative and postoperative complications are common. Recurrent disease is possible after all types of treatments, including fasciectomies.

Surgeons performing fasciectomies need to discuss potential complications and recurrence with their patients and set realistic expectations for efficacy and safety. Unfortunately, no concise source of estimated surgical complication rates exists. The purpose of this review is to provide a single resource of intraoperative and postoperative complications associated with fasciectomy for Dupuytren's disease.

## METHODS

### Identification of studies

Analysis of surgical complications was limited to those associated with fasciectomy and aponeuroectomy. To identify published, original research that reported surgical complications associated with surgery for Dupuytren's disease, a MEDLINE search was conducted with the following search parameters: fasciectomy[Title/Abstract] OR aponeurectomy[Title/Abstract] OR surgery[Title/Abstract] OR operate*[Title/Abstract] AND Dupuytren*[Title/Abstract] NOT review[Publication Type]. Search limitations included human subjects, English language, and dates of October 31, 1988, to October 31, 2008.

### Study selection

Search results were manually evaluated for relevance. Studies that did not report complication rates associated with fasciectomy or aponeurectomy were not included in the analysis. Studies that reported complication rates associated with fasciotomy, aponeurotomy, amputation, or postsurgical application of the S-Quattro external fixation device were excluded. Case studies were also excluded.

### Data analysis

Overall complication rates, complication rates according to types of disease (primary or recurrent disease), and complication rates according to time (intraoperative vs postoperative) and type were collated. Studies that did not specifically state whether patients had primary disease or recurrent disease were assumed to have had primary disease.

Averages and ranges were calculated for each complication described. The manner in which complications were reported varied from study to study (ie, by ray/finger; by hand; by patient); conversion of all surgical complication rates to a common denominator was not possible. Average rates were calculated and ranges were reported for each surgical complication across studies; the sum of all numerators was divided by the sum of all denominators and multiplied by 100.

## RESULTS

### Study attributes

A total of 143 articles were identified. One hundred two articles were excluded from the analysis (pathology, *n* = 16; treatment techniques, *n* = 17; postoperative care, *n* = 9; case studies, *n* = 13; long-term follow-up, *n* = 8; risk factors, *n* = 10; non-Dupuytren's disease, *n* = 12; surgery other than fasciectomy, *n* = 3; and other, *n* = 14). The remaining 41 articles met the inclusion criteria, reported complications associated with surgery for Dupuytren's disease, and were deemed appropriate for analysis: 27 evaluated primary (or otherwise not specified) disease, 2 evaluated recurrent disease, and 12 evaluated mixed populations (primary or recurrent disease) (Table 1).^4^,^10^,^27^,^30^,^32^,^39^^-^74 Of the 41 studies, 28 studies reported overall surgical complication rates ranging from 3.6% to 39.1%.

### Complications in patients with primary disease

Of the 27 studies that evaluated patients with primary disease,^10^,^32^,^47^^-^63,65^-^71,74 16 studies reported intraoperative complications. These complications included digital nerve injury (3.4%; range, 0.0%–7.7%) and digital artery injury (2.0%; range, 0.0%–2.6%) (Table 2).*

All 27 primary-disease studies reported postoperative complications,^10^,^32^,^47^^-^63,65^-^71,74 the most common being wound-healing complications (22.9%; range, 0.0%–86.0%), incisional scar pain (17.4%), dysesthesia/paresthesia (13.5%), hypoesthesia (10.1%; range, 6.0%–17.9%), flare reaction (9.9%; range, 2.1%–51.5%), reflex sympathetic dystrophy (5.8%; range, 0%–69.2%), infection (2.4%; range, 0–8.6%), and hematoma (2.1%; range, 0%–13%).

### Complications in patients with recurrent disease

Only 2 studies examined patients with recurrent disease exclusively. One study did not report intraoperative complications; the other evaluated intraoperative complications and reported no digital artery injuries (Table 3).^72^,^73^ Both studies reported postoperative complications: hyperesthesia (20.0%), local cold intolerance (20.0%), hypoesthesia (15.8%), and necrosis (11.1%). No cases of bleeding, infection, graft failure, or reflex sympathetic dystrophy were observed.

### Complications in mixed populations (primary and recurrent diseases combined)

Seven studies reported intraoperative complications in a mixed population (ie, primary and recurrent disease populations combined). The overall intraoperative complications in these studies were digital nerve injury (3.6%; range, 0.6%–7.8%), digital artery injury (3.3%; range, 0.8–9.7%), and tendon injury (0.02%) (Table 4).^4^,^27^,^30^,^40^,^44^,^46^,^64^

Eleven mixed-population studies reported overall postoperative complications: the most common were stiffness (15.4%; range, 1.6%–51.5%), hypoesthesia (14.0%), scar hypertrophy (10.0%), and scar contracture (9.4%).^4^,^27^,^30^,^39^^-^43,45,46,64

### Comparison of complications in patients with primary or recurrent disease

**Figure 1:**
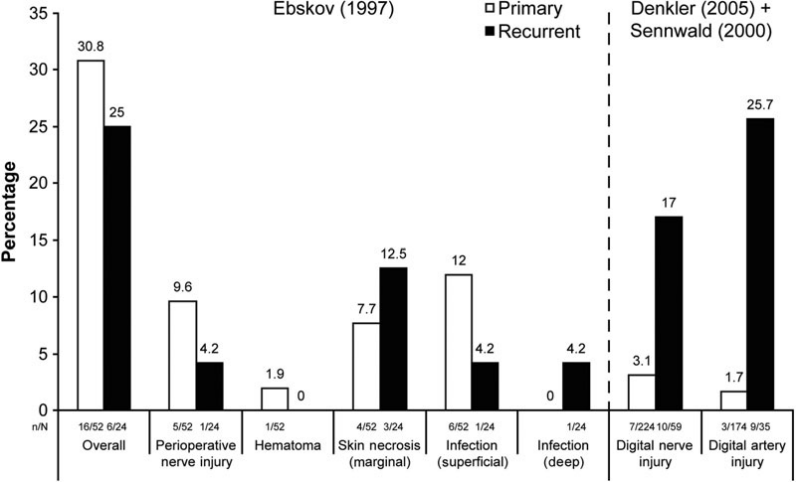
Surgical complications in studies (*n* = 3)^27^,^30^,^64^ that compared primary disease versus recurrent disease.

Three studies reported surgical complication rates separately for patients with primary disease and recurrent disease (Table 5 and Fig 1).^27^,^30^,^64^ Only one study reported overall complication rates, which were slightly higher in patients with primary disease (30.8%) than in those with recurrent disease (25.0%).^64^ Digital artery injury and digital nerve injury were more commonly observed in patients with recurrent disease than those with primary disease. The incidence of digital artery injury and digital nerve injury was 1.7% (3/174) and 3.1% (7/224), respectively, in patients with primary disease and 25.7% (9/35) and 17.0% (10/59), respectively, in patients with recurrent disease, indicating a approximately 10-fold difference (˜2% vs ˜20%) (Fig 1).^27^,^30^,^64^ However, the number of patients is too small for statistical significance.

## DISCUSSION

Data from this analysis clearly demonstrate that complications associated with fasciectomy for the treatment of patients with Dupuytren's disease are varied and relatively common. Data from studies that evaluated patients with primary disease showed that wound-healing complications and pain were most common. Conversely, patients with recurrent disease were more likely to experience varied types of sensory abnormalities (eg, hyperesthesia, cold intolerance, hypoesthesia) and necrosis. Data from the few studies that directly compared patients with primary and recurrent diseases showed that digital nerve injuries and digital artery injuries were much more common in patients with recurrent disease (typically ˜20%) than those with primary disease (typically ˜2%), although larger numbers of patients are needed for a valid statistical comparison. Pain was less common in patients with recurrent disease, perhaps because those who previously underwent fasciectomy and developed a pain-related complication were unlikely to undergo surgery a second time.

Surgical complication rates in the present analysis were physician reported. A large patient survey study (*N* = 1177) conducted by the British Society for Surgery of the Hand provides insight into patient-reported complications after Dupuytren's surgery.^75^ Patients with Dupuytren's disease were identified by hand surgeons throughout the United Kingdom and were invited to complete a questionnaire about surgical outcomes and complications. Patients' self-reported complications were 35.8% for numbness and 19.8% for infection.^75^ These values are much higher than the physician-reported complications rates provided in the current analysis.^75^

As with all surgeries, complication rates generally correlate with invasiveness of the procedure. Patients with severe disease often have greater tissue involvement and require more complex measures to correct the finger deformity. Consequently, patients with severe disease at the time of surgery tend to experience more complications postfasciectomy.^46^,^75^ A retrospective analysis of 253 patients with Dupuytren's disease who underwent fasciectomy showed that complication rates increased with the severity of disease, particularly when PIP joint contracture was more than 60°.^46^ Dias and Braybrooke^75^ made a similar observation, showing a clear relationship between the incidence of self-reported complications and the severity of the initial deformity, with patients who had severe disease at the time of surgery reporting more surgical complications. Loos et al^4^ in a large study of almost 3000 patients noted a statistically significant correlation between worsening stage of the disease and postoperative complications.

Several limitations of the present analysis must be taken into consideration when interpreting the data. First, the manner in which complication rates were reported varied from study to study and included complications per ray or finger, per patient, and per hand. Conversion of complication rates to one common denominator was not possible, so the overall rates and ranges represent blended data. Given the large number of studies (*n* = 41) included in the analysis, overall interpretation should not be affected, though this limitation may explain why the ranges associated with some complications are relatively broad. Second, several factors, such as patient diathesis, baseline disease severity, the type of joint affected (ie, MP or PIP), and multiple digit involvement, that can impact the frequency of surgical complications were not analyzed separately. Complication rates in the present analysis are therefore based on a heterogeneous patient population and cannot be directly compared with a specific patient subset.

In the absence of an approved pharmacotherapy, surgery provides the best opportunity for long-term functional improvement for patients with Dupuytren's disease. Although complete restoration of hand function is unlikely, most patients will experience significant gain in function. However, several drawbacks to surgery exist. First, surgery does not cure Dupuytren's disease and recurrences rates are high, ranging from 26% to 80%.^37^ Second, surgery in patients with recurrent disease is usually more challenging because scarring and anatomic distortion from prior procedure(s) increases the likelihood of neurovascular complications. Third, rehabilitation after open surgery may be prolonged. Finally, multiple, repetitive surgical procedures have their limitations and not all patients are good candidates for surgery.

## CONCLUSIONS

This is the first report to extensively collect and analyze complications associated with surgery for Dupuytren's disease in clinical practice. Data from this study indicate that complications of surgery not only occur frequently but are also varied. Therefore, surgeons who perform fasciectomies for Dupuytren's disease should be mindful of the potential for intraoperative and postoperative complications and should counsel their patients accordingly. Furthermore, the severity of the disease and surgical history of the patient should be considered when anticipating complications. Patients undergoing fasciectomy for recurrent disease are more likely to experience either digital nerve injury or digital artery injury than patients with primary disease.

In conclusion, results of this study underscore the importance of treating Dupuytren's as an incurable genetic disease understanding that surgical excision, fasciectomy, has a high rate of major and minor complications. Surgeons must understand that while fasciectomy for Dupuytren's does offer a chance at long-term “straight” fingers, there is a high cost in terms of numbers of complications that are borne by the patient.

## Acknowledgments

The author thank Maribeth Bogush, PhD, and Lynn Brown, PhD, for editorial assistance.

## Figures and Tables

**Table 1:** Studies included in the analysis*

No.	Authors (year)	Study design	No. of patients	No. of hands	No. of joints	Joint type	Follow-up period	Surgical techniques	Disease category
1	Srivastava et al (1989)^39^	Retrospective	10	12	NR	MP, PIP	1–10 y	Fasciectomy, limited or radical; Z-plasty closure or open-palm technique; amputation for advanced disease	Mixed: Primary, 70%; recurrent, 30%
2	Sennwald (1990)^27^	Retrospective	98	103	NR; 239 rays	NR	3–6 mo	Fasciectomy, radical; rotation flap or Z-plasty	Mixed: Primary, 74.8%; recurrent, 25.2%
3	Moermans (1991)^40^	Prospective	175	213	503	MP, PIP	Mean, 2.6 ± 1.6 y; range, 0–7 y	Aponeurectomy, segmental	Mixed: Primary, 83.1%; recurrent, 16.9%
4	Foucher et al (1992)^41^	Retrospective	107	NR	NR; 140 digits	MP, PIP	>5 y	Fasciectomy, limited; open-palm technique and/or digit	Mixed: Primary, 95%; recurrent, 5%
5	Searle and Logan (1992)^42^	Retrospective	32	NR	NR; 40 rays	NR	Mean, 38 mo; ≥24 mo	Dermofasciectomy	Mixed: Primary, 53%; recurrent, 47%
6	Beyermann et al (2004)^43^	Prospective	43	43	43	PIP	24 wk	Fasciectomy (*n* = 32), with CLM release (*n* = 11)	Mixed: Primary, 67.4%; recurrent, 32.6%
7	Meathrel and Thoma (2004)^44^	Retrospective	149	NR	NR; 261 digits	NR	NR	Fasciectomy, palmar	Mixed: Primary, 87.2%; recurrent, 12.8%
8	Kobus et al (2007)^45^	Retrospective	253	287	NR	MP, PIP	Mean, 3 y	Fasciectomy, radical, with V-Y–plasty	Mixed: Primary, 86.2%; recurrent, 13.8%
9	Loos et al (2007)^4^	Retrospective	2919	4388	NR	MP, PIP, DIP	NR; data span 50-y period	Fasciectomy, limited (94.8% of procedures) or total; amputation	Mixed: Primary, 88%; recurrent, 12%; data not complete
10	Bulstrode et al (2005)^46^	Retrospective	253	NR	NR	NR	Mean, 3.6 y; range, 9 mo–11 y	Fasciectomy, modified Skoog's technique	Mixed: Primary, 75.5% (191/253); recurrent, 24.5% (62/253)
11	Ebskov et al (1997)^64^	Prospective	76	NR	NR; mean rays involved: primary, 2.1; recurrent, 1.8	MP, PIP	3 wk	Fasciectomy, radical, open-palm technique	Mixed: Primary, 68.4%; recurrent, 31.6%
12	Denkler (2005)^30^	Retrospective	Hospital, 26; office, 40	NR	Hospital, 73; office, 93; digits: hospital, 42; office, 60	MP, PIP	Hospital: mean, 10.6 ± 21.9 mo; median, 3 mo; office: 9.3 ± 9.5 mo; median, 4 mo	Fasciectomy, hospital group (traditional anesthetics with tourniquet; 43 digits) vs office group (local anesthetics with epinephrine and no tourniquet; 60 digits)	Mixed: Hospital: primary, 88.5% (23/26); 11.5% (3/26); office: primary, 95.0% (38/40); 5.0% (2/40)
13	Andrew and Kay (1991)^47^	Prospective	46	50	79	MP, PIP	12 mo	Aponeurectomy, segmental	Primary, 100%
14	Liu and Chen (1991)^48^	Retrospective	27	32	NR; 58 digits	NR	Mean, 5.3 y; range, 1–16 y	Fasciectomy with longitudinal, lazy-s, zigzag, or transverse incision	Primary, 100%
15	Robins et al (1993)^49^	Prospective	50	50	NR	NR	NR	Fasciectomy, local; usually with zigzag incision	Primary, 100%
16	Cools and Verstreken (1994)^50^	Retrospective	28	33	NR	MP, PIP	Mean, 2.5 y	Fasciectomy, partial; open-palm technique	Primary, 100%
17	Citron and Nunez (2005)^51^	Prospective	79	79	NR	MP, PIP	≥2 y	Fasciectomy, modified Bruner incision (*n* = 47) vs longitudinal incision with Z-plasty closure (*n* = 33)	Primary, 100%
18	Van Giffen et al (2006)^52^	Retrospective	38	38	63 (fifth ray only)	MP, PIP	Mean, 54 mo; range, 27–75 mo	Fasciectomy, isolated limited or segmental; dermofasciectomy	Primary, 100%
19	van Rijssen et al (2006)^32^	Prospective	113	117	127†	MP, PIP, DIP	6 wk	Limited fasciectomy	Primary, 100%
20	Skoff (2004)^53^	Prospective	30	NR	NR	MP, PIP	Synthesis: mean, 2.7 y; range, 2.0–3.0 y; open-palm technique: mean, 3.5 y; range, 3.1–4.0 y	Fasciectomy, “synthesis” technique (*n* = 20) vs open-palm technique (*n* = 10)	Primary, 100%
21	Ritchie et al (2004)^54^	Prospective	14	19	19	PIP	Mean, 36 mo; range, 35–39 mo	Fasciectomy (8 little fingers), with CLM release (11 little fingers)	Primary, 100%
22	Misra et al (2007)^55^	Prospective	35	NR	52	MP, PIP	Mean, 1.5 y; range, 1–3 y	Fasciectomy with Z-plasty (19 joints) ± PIP joint release (33 joints)	Primary, 100%
23	Sorene et al (2007)^56^	Retrospective	19	22	44	IP, MP, PIP, DIP	Mean, 30 mo; range, 12–118 mo	Fasciectomy, selective, through modified Bruner palmodigital incisions	Primary, 100%
24	Stahl and Calif (2008)^57^	Retrospective	23	26	NR	MP, PIP, DIP	Mean, 2.5 y; range, 1.5–19 y	Fasciectomy, limited, through zigzag digitopalmar incision ± CLM release of PIP joint	NR
25	Vigroux and Valentin (1992)^58^	Retrospective	56	76	NR; 137 digits	MP, PIP	Mean, 12 y, 7 mo; range, 10–22 y	Fasciectomy, regional ± PIP capsulectomy	NR
26	Foucher et al (1995)^59^	Retrospective	54	NR	NR; 67 digits	MP, PIP	Mean, 6.6 y; ≥5 y	Fasciectomy, open-palm technique	NR
27	De Maglio et al (1996)^60^	Retrospective	124	145	NR	MP, PIP	Mean, 33 mo; range, 6–59 mo	Aponeurectomy, selective; Skoog's and/or Dieckman/Iselin routes of access	NR
28	Shaw et al (1996)^61^	Retrospective	25	26	NR; 39 digits	MP, PIP	9–19 y	Fasciectomy, palmar; open-palm technique	NR
29	Weinzweig et al (1996)^62^	Retrospective	28	42	42	PIP	Mean, F, 10.1 mo; F + C, 6.4 mo	Fasciectomy (18 patients, 27 joints); F + C; 10 patients, 15 joints)	NR
30	Citron and Messina (1998)^63^	Retrospective	13	NR	NR; 18 digits	PIP	Mean, 18 mo; range, 2–30 mo	Preoperative traction + fasciectomy ± fasciotomy	NR
31	Gonzalez et al (1998)^74^	Retrospective	16	19	40	IP, MP, PIP	Mean, 25 mo; range, 6–168 mo	Fasciectomy, selective, with Z-plasty; fasciectomy, segmental, with multiple curvilinear incisions or Z-plasty	NR
32	Clibbon and Logan (2001)^65^	Retrospective	56	67	67	MP	Mean, 30 mo; range, 12–86 mo	Aponeurectomy, segmental (palmar)	NR
33	Evans et al (2002)^66^	Retrospective (1983–1993; TA only); prospective (1993–1999, TA and NTA)	268	NR	NR; mean number of digits undergoing surgery: 1.96 (TA); 1.6 (NTA)‡	MP, PIP	NR	Fasciectomy, with TA (*n* = 103) or NTA (*n* = 165)	NR
34	Barr et al (2003)^67^	Retrospective	5	5	14	MP, PIP	Mean, 14 mo; range, 3–34 mo	Fasciectomy, with Z-plasty + intramuscular tenotomy of flexor digitorum superficialis in distal forearm	NR
35	Abe et al (2004)^68^	Retrospective	57	73	146	IP, MP, PIP	Mean, 4 y; range, 2–17 y	Fasciectomy, subtotal	NR
36	Ali et al (2006)^69^	Retrospective	32	35	NR	NR	Mean, 6 mo; range, 2–13	Fasciectomy, selective regional; ulnar-based skin flap	NR
37	Coert et al (2006)^10^	Retrospective	261 (558 operations)	556		MP, PIP, DIP	Mean, 7.3±0.44 y; range, 0.3–48 y	Fasciectomy, partial	NR; average number of operations was 2.54 per patient over 8-y study period
38	Reuben et al (2006)^70^	Prospective	300	NR	NR	NR	1, 3, 12 mo postsurgery	Fasciectomy, with general anesthesia, axillary block, or intravenous regional anesthesia with lidocaine ± clonidine	NR
39	Anwar et al (2007)^71^	Retrospective	657; 109 women, 548 men	119 women, 589 men	123 women, 760 men	MP, PIP, DIP	NR	Fasciectomy, fasciectomy + local flap, dermofasciectomy	NR
40	Ekerot (1995)^72^	Retrospective	15	16	NR; 17 flaps	MP, PIP	≤2 y	Fasciectomy, radical, with distally based dorsal hand flap; PIP joint capsulectomy in 4 fingers	Recurrent, 100%
41	Roush and Stern (2000)^73^	Retrospective	19	NR	NR; 28 digits	MP, PIP, DIP	Median, 4 y; range, 1–15 y	Fasciectomy, limited, and IP arthrodesis; dermofasciectomy; fasciectomy and local flaps	Recurrent, 100%

*NR indicates not reported; TA, tension applied; NTA, no tension applied; MP, metacarpophalangeal; PIP, proximal interphalangeal; DIP; distal interphalangeal; IP, interphalangeal; fasciectomy + capsulotomy; capsuloligamentous.

†An additional 150 joints were treated with percutaneous needle fasciotomy but were excluded from this analysis.

‡An additional 150 finger joints were treated with percutaneous needle fasciotomy but were excluded from this study since this study is discussingcomplications of surgical fasciectomy (excsion) for Dupuytren's.

**Table 2:** Reported complications* of surgery for primary Dupuytren's disease

Complication	No. of studies reporting complications	Average, % (*n*/*N*)	Range, %
Intraoperative			
Digital artery injury^10^,^52^,^54^,^71^	4	2.0 (20/989)	0–2.6
Digital nerve injury†	15	3.4 (51/1510)	0–7.7
Postoperative			
Amputation (classified as postoperative complication)^10^	1	1.5 (4/261)	…
Carpal tunnel syndrome^56^,^62^	2	6.4 (3/47)	3.6–10.5
Clinodactyly^50^	1	3.0 (1/33)	…
Complex regional pain syndrome (see “reflex sympathetic dystrophy”)		…	…
Contracture^48^,^63^	2	6.7 (3/45)	6.2–7.7
Dysesthesia or paresthesia^32^,^59^	2	13.5 (15/111)	3.7–22.8
Edema^62^	1	7.1 (2/28)	…
Flare reaction^66^,^71^	2	9.9 (92/925)	2.1–51.5
Flexion, loss of^47^,^49^	2	4.2 (4/96)	4.0–4.3
Hematoma^32^,^48^^-^50,55,57,59,68,70	9	2.1 (14/657)	0–13.0
Hyperesthesia^50^	1	3.0 (1/33)	…
Hypoesthesia^50^,^52^,^62^	3	10.1 (10/99)	6.0–17.9
Incisional scar pain^57^	1	17.4 (4/23)	…
Infection‡	19	2.4 (44/1860)	0–8.6
Necrosis (skin, flap, or graft)^10^,^49^,^50^,^52^,^53^,^59^,^60^,^62^,^68^,^69^	10	4.3 (31/713)	0–10
Pain (not otherwise specified)^50^,^59^	2	13.8 (12/87)	3–20.3
Reflex sympathetic dystrophy (complex regional pain syndrome)^10^,^49^^-^53,57^-^63,65,70,71	16	5.8 (106/1828)	0–69.2
Stiffness^62^	1	3.6 (1/28)	…
Swan neck deformity^54^	1	7.1 (1/14)	…
Tenosynovitis^50^	1	3.0 (1/33)	…
“Trigger finger”^56^	1	5.3 (1/19)	…
Wound-healing complication^32^,^47^,^49^,^58^,^60^,^62^,^66^,^67^∥	8	22.9 (145/634)	0–86.0.

*Studies that reported no cases of a particular complication were included in calculations.

†*References 10, 32, 47, 49, 51, 53-55, 57, 61, 62, 65, 68, 71*.

‡*References 10, 32, 48-50, 52-56, 60, 62, 65, 67-71, 74*.

§Includes cases of algodystrophy.

∥One study reported no wound dehiscence.

**Table 3:** Reported complications* of surgery for recurrent Dupuytren's disease

Complication	No. of studies reporting complication	Average, % (*n*/*N*)
Intraoperative		
Digital artery injury^73^ (anesthetic)	1	0 (0/19)
Postoperative		
Bleeding^72^	1	0 (0/17)
Graft failure^73^	1	0 (0/19)
Hyperesthesia^72^	1	20.0 (3/15)
Hypoesthesia^73^; poor to fair numbness noted postoperatively	1	15.8 (3/19)
Infection^72^,^73^	2	0 (0/36)
Necrosis (skin, flap, or graft)^72^,^73^	2	11.1 (4/36)
Reflex sympathetic dystrophy (complex regional pain syndrome)^73^	1	0 (0/19)
Local cold intolerance^72^	1	20.0 (3/15)

*Studies that reported no cases of a particular complication were included in calculations.

**Table 4:** Reported complications* of surgery for primary and recurrent Dupuytren's diseases (mixed populations)

Complication	No. of studies reporting complication	Average, % (*n*/*N*)	Range, %
Intraoperative			
Digital artery injury^27^,^30^,^46^	3	3.3 (14/422)	0.8–9.7
Digital nerve injury^4^,^27^,^30^,^40^,^44^,^46^,^64^	7	3.6 (135/3779)	0.6–7.8
Tendon injury^4^	1	0.2 (5/2919)	…
Postoperative			
Bleeding^4^	1	1.2 (35/2919)	…
Complex regional pain syndrome (see “reflex sympathetic dystrophy”)			
Carpal tunnel syndrome^46^	1	0.8 (2/253)	…
Severe dysesthesia leading to amputation^27^	1	1.0 (1/103)	…
Flexion, loss of^30^	1	1.5 (1/66)	…
Graft failure leading to amputation^42^	1	3.1 (1/32)	…
Hematoma^27^,^30^,^40^,^46^,^64^	5	1.8 (13/711)	1.3–2.9
Hypoesthesia^43^	1	14.0 (6/43)	…
Infection^4^,^27^,^30^,^46^,^64^	5	3.9 (134/3424)	0.9–10.5
Necrosis (skin, flap, or graft)^4^,^30^,^40^,^45^,^46^,^64^	6	2.5 (93/3780)	0–9.2
Transient paralysis^27^†	1	0.9 (1/103)	…
Reflex sympathetic dystrophy (complex regional pain syndrome)^27^,^40^,^41^,^46^,^64^	5	4.5 (34/752)	0–18.4
Scar contracture from graft^42^	1	9.4 (3/32)	…
Scar hypertrophy^39^	1	10.0 (1/10)	…
Stiffness^27^,^45^	2	15.4 (55/356)	1.6–51.5
Vascular damage^45^	1	0.8 (2/253)	…
Wound dehiscence^30^	1	4.5 (3/66)	…
Wound-healing complications such as skin edge necrosis or slough^46^	1	1.2 (3/253)	…

*Studies that reported no cases of a particular complication were included in calculations.

†Transient paralysis assumed to be caused by a tourniquet.

**Table 5:** Intrastudy comparison of surgical complications* in patients with primary or recurrent Dupuytren's disease

Complication	Primary, % (*n*/*N*)	Recurrent, % (*n*/*N*)
Overall	30.8 (16/52)^64^	25.0 (6/24)^64^
Digital nerve injury	1.3 (1/77)^27^	26.9 (7/26)^27^
	1.5 (1/95)^30^	22.2 (2/9)^30^
	9.6 (5/52)^64^	4.2 (1/24)^64^
Digital artery injury	2.6 (2/77)^27^	30.8 (8/26)^27^
	1.0 (1/97)^30^	11.1 (1/9)^30^
Hematoma	1.9 (1/52)^64^	0 (0/24)^64^
	0 (0/77)^27^	7.7 (2/26)^27^
Skin necrosis (marginal)	7.7 (4/52)^64^	12.5 (3/24)^64^
Infection (superficial)	12.0 (6/52)^64^	4.2 (1/24)^64^
Infection (deep joint infection that led to amputation)	0 (0/52)^64^	4.2 (1/24)^64^

*Studies that reported no cases of a particular complication were included in calculations.

## Footnotes

**References 10, 32, 47, 49, 51-55, 57, 58, 61, 62, 65, 68, 71*.
